# Real-word evaluation of differences in bowel preparation for colonoscopy between the digestive and the non-digestive physicians: A retrospective study

**DOI:** 10.3389/fgstr.2022.946459

**Published:** 2022-07-28

**Authors:** Cenqin Liu, Xin Yuan, Hui Gao, Zhixin Zhang, Weihong Wang, Jiarong Xie, Hongpeng Lu, Jian Chen, Chaohui Yu, Lei Xu

**Affiliations:** ^1^ Department of Gastroenterology, Ningbo Hospital, Zhejiang University, Ningbo, China; ^2^ Department of Gastroenterology, The First Affiliated Hospital, College of Medicine, Zhejiang University, Hangzhou, China; ^3^ Department of Gastroenterology, Ningbo First Hospital, Ningbo, China; ^4^ College of Medicine, Ningbo University, Ningbo, China; ^5^ Department of Gastroenterology, Xiangshan County Red Cross Taiwan Compatriots Hospital, Ningbo, China

**Keywords:** bowel preparation, colonoscopy, physicians, predictive factors, real-word

## Abstract

**Introduction:**

Using real-world data, we compared the quality of bowel preparation instructed by the digestive and non-digestive physicians in outpatients for colonoscopy and identified potential risk factors.

**Methods:**

This was a retrospective study based on real-world data, which were collected from the Ningbo First Hospital in China from December 2019 to October 2020. Outpatients included were classified into the digestive and the non-digestive physician groups according to the referring physician. The primary outcome was adequate bowel preparation measured by the Boston Bowel Preparation Scale (BBPS), namely, a BBPS score of 2 or higher in any colonic segment and a total score ≥ 6. Secondary outcomes included the total mean BBPS scores and possible risk factors associated with poor bowel preparation.

**Results:**

There were 671 outpatients included, with 392 in the digestive physician group and 279 in the non-digestive physician group. Adequate bowel preparation was 84.2% in the digestive physician group and 71.0% in the non-digestive physician group (odds ratio [OR]: 1.50, *p* < 0.001), and the latter had lower total mean BBPS scores (6.12 ± 1.33 vs. 6.66 ± 1.29, *p* < 0.001). The non-digestive physician was an independent risk factor according to the multivariate logistic regression analysis (OR: 0.45, *p* < 0.001).

**Conclusion:**

The quality of bowel preparations instructed by non-digestive physicians was inferior to digestive physicians, which was a factor potentially associated with poor bowel preparation (ClinicalTrials.gov number: NCT04738578).

## Introduction

Colorectal cancer (CRC) is one of the world’s leading malignant cancers, ranking third in terms of incidence and second in terms of mortality (1). Polypectomy could significantly reduce CRC incidence (2). However, inadequate bowel preparation has a detrimental effect on all aspects of the colonoscopy procedure (3), including a reduction in the detection rates of adenomas (4, 5) and colonoscopy surveillance intervals (6–8) and an increase in the procedural time, potential adverse event rates, and healthcare costs (9, 10).

Although, more and more risk factors were identified to be associated with the poor bowel preparation, including patients’ baseline characteristics, clinical conditions, medication use, and hospitalization status (11–13). Inadequate bowel cleansing had still been reported in up to 13.3%–35% of patients undergoing colonoscopy (6, 14), which necessitates repeat procedures or results in unsatisfactory diagnostic accuracy.

In China, outpatients with different cardinal symptoms would make an appointment with different referring physicians. Therefore, the colonoscope examination would be scheduled and the bowel preparation would be instructed by their referring physicians at this time if a patient wants to do a colonoscopy incidentally for screening or diagnosis. On this occasion, referring physicians were involved in recognizing the indications for colonoscopy and played an important role in patient education and bowel preparation procedures. However, most studies mainly focused on patient factors such as sex, age, body mass index (BMI), disease status, diet, and low socioeconomic status (15–17) to hammer out a solution. Few studies focused on physicians to explore the possible risk factors. Non-digestive referring physicians might not be able to achieve equivalent adequate bowel cleansing due to their relatively poor knowledge of colonoscopy and bowel preparation.

Therefore, we aimed to evaluate the quality of bowel preparation in outpatients who were instructed by digestive physicians or non-digestive physicians and try to identify the new risk factors for suboptimal intestinal preparation.

## Method

### Study design

This was a retrospective study based on the real-world data, which were collected beforehand by research assistants who were unaware of the trial from the endoscopy room of the First Hospital of Ningbo (Ningbo, China) from December 2019 to October 2020. This study was approved by the local institutional review boards of the centers involved in the trial and was registered at ClinicalTrials.gov on 02/01/2021 (NCT04738578).

### Patients and procedure

All outpatients who underwent unsedated colonoscopy and the endoscopic video transcribed from December 2019 to October 2020 were included. The exclusion criteria were as follows: (1) patients whose referring doctor could not be searched, (2) missing Boston Bowel Preparation Scale (BBPS) score and baseline characteristics, (3) colonic segments were incomplete or failed colonoscopy examination that did not reach the cecum except for the poor bowel preparation, and (4) examinations of hospitalized patients. All patients underwent a pre-colonoscopy bowel cleanse with the polyethylene glycol solution. A uniform paper manual on general considerations of bowel preparation was distributed to every outpatient, which included a low-residue diet that avoids foods containing seeds and other indigestible substances. A verbal, detailed explanation to patients face to face would be provided as needed according to the referring physicians’ practice. Then, research assistants obtained and recorded the patients’ baseline characteristics including sex, age, BMI, low-residue diet, constipation, previous history of abdominal surgery, complications, and so forth on the day of their procedure. Personal comorbidities were categorized as cardiovascular disease, metabolic disease, digestive disease, and neurologic disease. Hypertension, hypotension, and coronary heart disease were included in the analysis of cardiovascular disease due to the sample size. Similarly, diabetes, hyperlipidemia, and hyperuricemia were included in the analysis of metabolic disease; hepatocirrhosis and inflammatory bowel disease were included in the analysis of digestive disease; and neurologic disease only included stroke. The BBPS score was recorded by another data collector with an operator who was blinded to the identities of the referring doctors (18, 19). All colonoscopies were performed by experienced endoscopists from the gastroenterology department. Finally, the researchers reviewed the endoscopic video and scored again on bowel cleanliness. Outpatients were divided into two groups according to their referring physicians: digestive physicians and non-digestive physicians. In this center, digestive physicians were also the endoscopic technicians, being responsible for digestive disease diagnosis and endoscopic treatment. Non-digestive physicians were defined as those who primarily specialized in other clinical categories of disease or did not perform colonoscopies. Outpatients’ medical information, including referring physicians, was retrieved and recorded from a computerized database.

### Outcomes

The bowel cleanse was evaluated on the BBPS score, which scored on each colonic segment ranging from 0 to 3; the higher the BBPS score is, the better the bowel preparation quality. The primary outcome was the proportion of adequate bowel preparation (ABP) with a score of 2 or higher in any colonic segment and a total BBPS score ≥ 6. The secondary outcomes included the mean BBPS scores, the proportion of the qualified segmental score of ≥ 2, and the possible factors associated with inadequate bowel preparation.

### Statistical analysis

Chi-squared test or Fisher’s exact test was used for categorical data, and Student’s t-test (comparison of means) was used for continuous variables. Single-factor and multivariate logistic regression analyses were performed to determine independent risk factors that influence the quality of bowel preparation. A two-sided *P* < 0.05 was considered to indicate a significant difference, and all statistical analyses were performed using SPSS (version 22, SPSS).

## Results

### Patient characteristics

**Figure 1:**
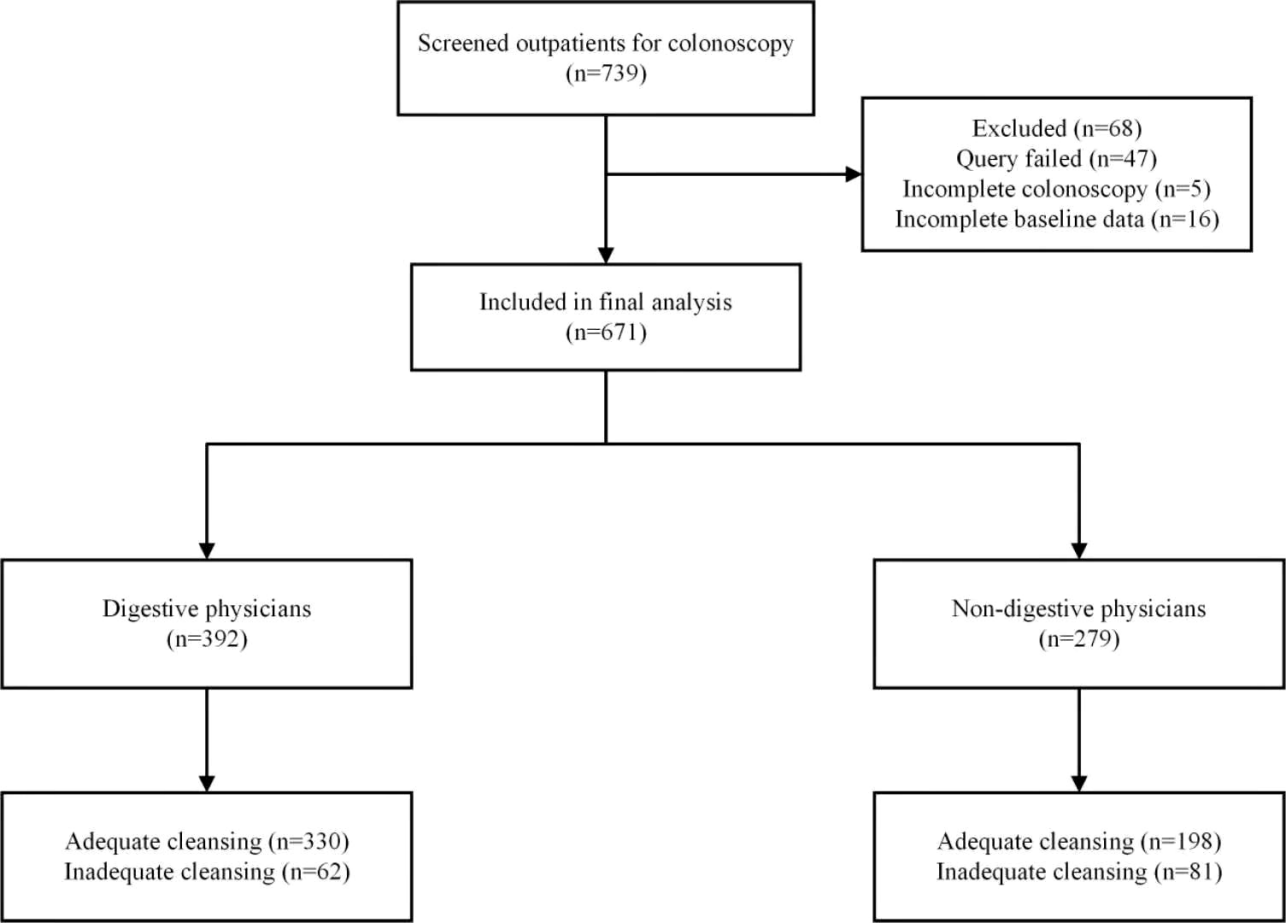
Study flowchart.

A total of 739 cases were collected; the referring doctor could not be identified for 47 patients, complete baseline characteristics were lacking for 16 patients, and a critical absence of information on the primary outcome was noted for five patients. A total of 671 outpatients with complete information were eligible and ultimately enrolled in the study. We classified outpatients into the digestive physician group (*n* = 392) and the non-digestive physician group (*n* = 279) (Figure 1). There was no significant difference in terms of sex, age, BMI, history of abdominal operation, complications, low-residue diet, or constipation for colonoscopy between the groups. The baseline characteristics of the subjects are shown in Table 1.

**Table 1:** Baseline characteristics of the study participants.

Characteristic	Digestive physiciann(*n*=392)	Non-digestive physician(*n*=279)	*P*-value
Sex, *n* (%)			
Male	189 (48.2)	133 (47.7)	0.889
Female	203 (51.8)	146 (52.3)	
Age, mean ± SD (years)	52.7 ± 13.8	51.8 ± 13.1	0.402
BMI, mean ± SD (kg/m^2^)	22.8 ± 3.2	23.3 ± 3.9	0.063
History of abdominal			
operation, *n* (%)	140 (35.7)	91 (32.6)	0.405
Comorbidity, *n* (%)			
Cardiovascular disease	98 (25.0)	59 (21.2)	0.255
Metabolic disease	56 (14.3)	46 (16.5)	0.422
Digestive disease	8 (2.0)	6 (2.2)	>0.999
Neurologic disease	4 (1.0)	2 (0.7)	>0.999
Constipation	72 (18.4)	50 (17.9)	0.883
Low-residue diet	319 (81.4)	214 (76.7)	0.140

Data are presented as the mean ± standard deviation or number (percentage) as appropriate; SD, standard deviation; BMI, body mass index.

### Outcome

**Figure 2:**
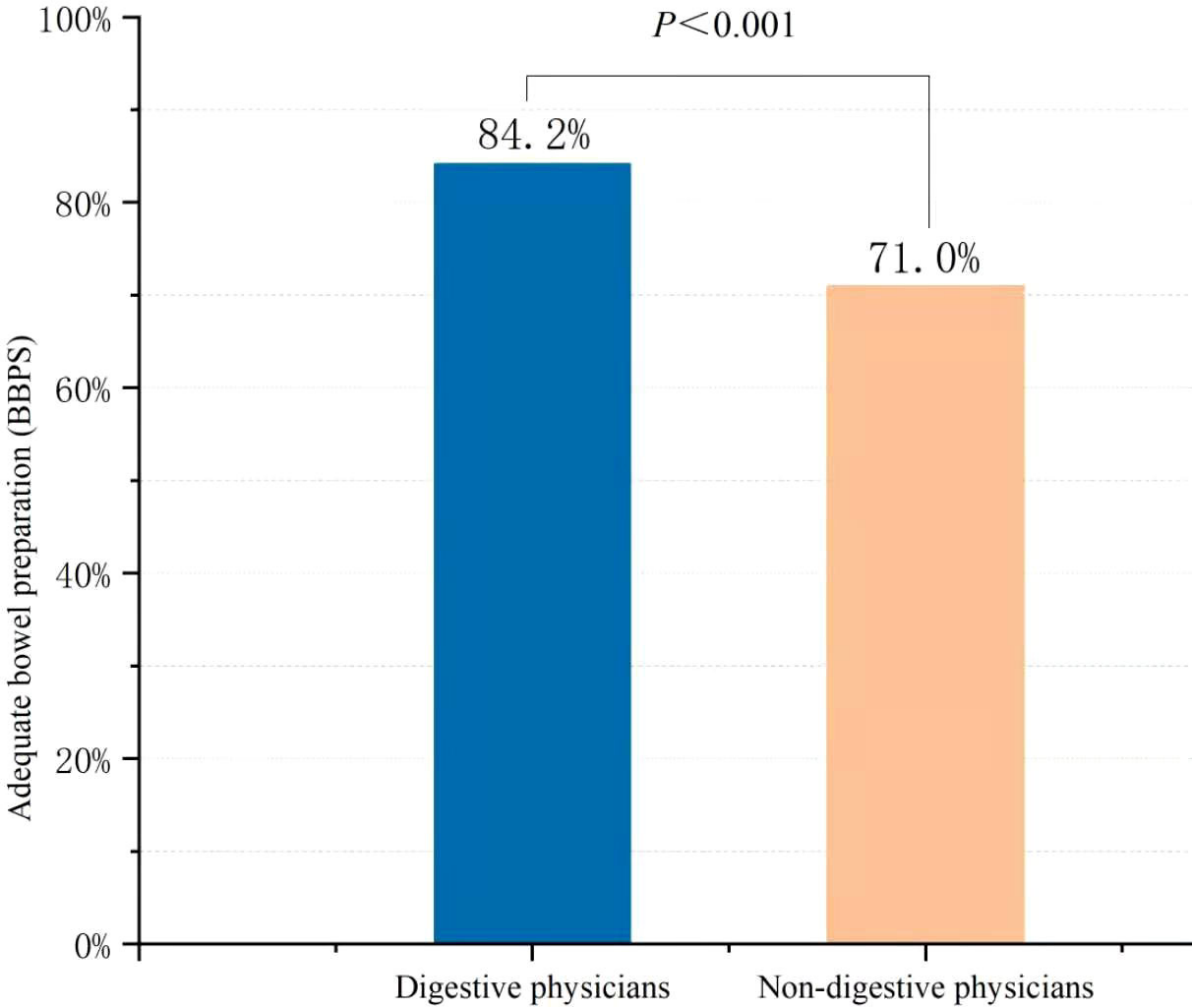
The rate of adequate bowel preparation in digestive physicians and non-digestive physicians among outpatients.

**Figure 3:**
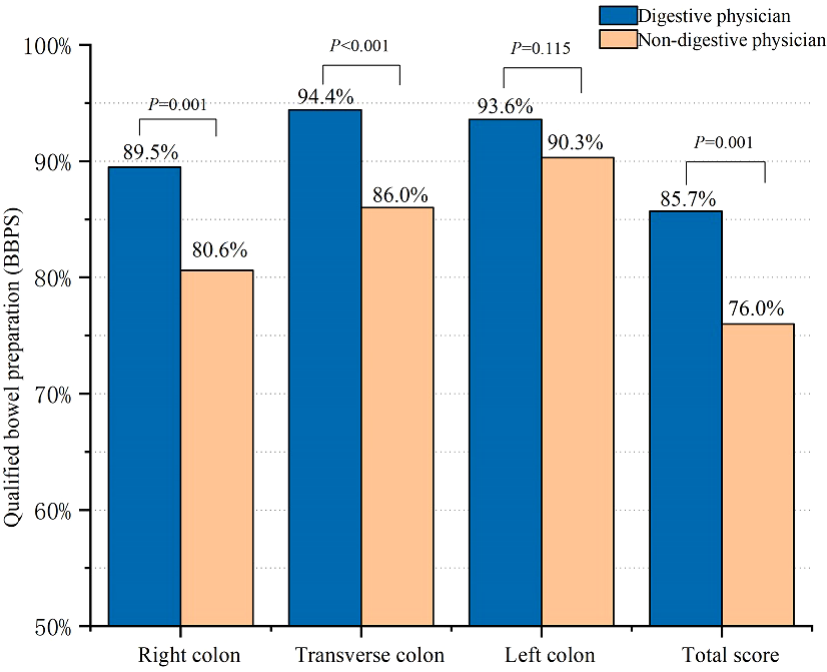
The rate of qualified bowel preparation in digestive physicians and non-digestive physicians among outpatients.

**Figure 4:**
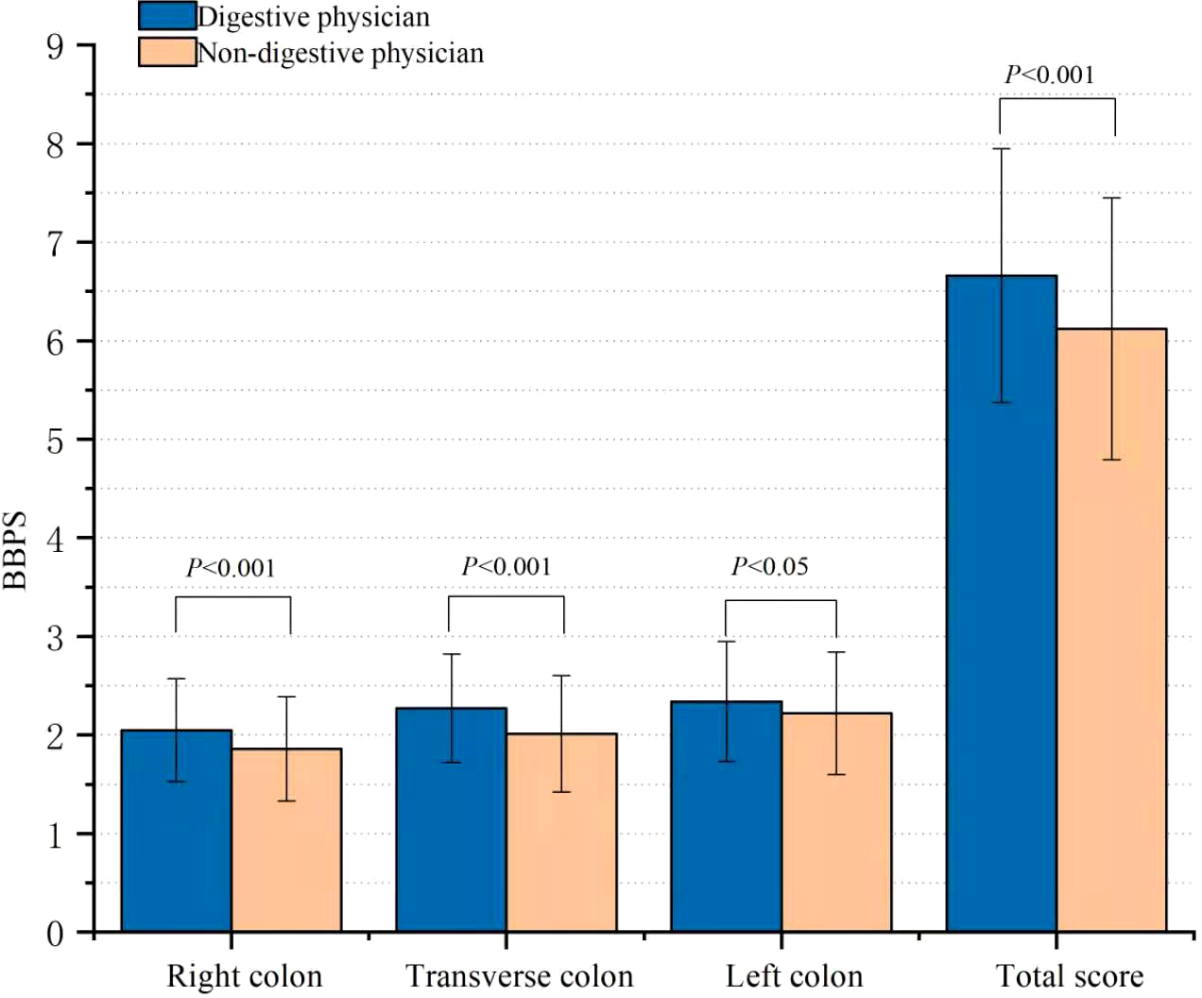
Comparison of the mean BBPS score.

The percentage of ABP in the digestive physician group was significantly higher than that in the non-digestive physician group (84.2% vs. 71.0%, *p* < 0.001) (Figure 2). Outpatients instructed by the non-digestive physicians had lower qualified rates of the total BBPS scores (BBPS ≥ 6, 76.0% vs. 85.7%, *p* < 0.001) and lower scores for the right colon (BBPS ≥ 2 80.6% vs. 89.5%, *p* = 0.001), transverse colon (86.0% vs. 94.4%, *p* < 0.001), and left colon (90.3% vs. 93.6%, *p* = 0.115) (Figure 3). Both the mean of the total BBPS score and the segmental BBPS scores of the right colon and left colon were lower in the non-digestive physician group than in the digestive physician group (Figure 4).

Univariate logistic regression was used to identify the correlative factor associated with ABP (Table 2), and multivariate analysis was performed when the *P*-value was ≤ 0.1. The independent risk predictors of inadequate bowel preparation by multivariate logistic regression analysis were shown to be non-digestive physicians (*p* < 0.001) and BMI (*p* = 0.017), and the independent protective factor was having a low-residue diet.

**Table 2:** Analysis of risk factors associated with inadequate bowel cleansing.

Characteristic	Univariate analysis, *P*			Multivariate analysis, *P*		
	OR	95%CI	*P*	OR	95%CI	*P*
Non-digestive physician	0.46	0.32-0.67	<0.001	0.45	0.31-0.66	<0.001
Sex	1.06	0.73-1.53	0.759			
Age	1.01	1.00-1.02	0.184			
BMI	0.94	0.88-1.00	0.034	0.93	0.87-0.99	0.017
History of abdominal operation	0.93	0.63-1.37	0.725			
Comorbidity						
Cardiovascular disease	0.97	0.63-1.50	0.904			
Metabolic disease	0.60	0.37-0.96	0.031	1.69	0.68-4.23	0.267
Digestive disease	1.64	0.36-7.41	0.702			
Neurologic disease	0.27	0.05-1.34	0.108			
Constipation	1.51	1.00-2.44	0.052	1.58	0.99-2.52	0.057
Low-residue diet	0.49	0.32-0.74	0.001	0.50	0.32-0.77	0.002

CI, confidence interval; OR, odds ratio; BMI, body mass index.

## Discussion

Our study revealed that the quality of intestinal preparations for colonoscopy instructed by non-digestive physicians was significantly inferior to that instructed by digestive physicians. Instructed by the non-digestive physicians was a risk factor associated with the poor bowel preparation of outpatients. To the best of our knowledge, this study is the few to identify non-digestive physicians as a novel risk factor that had a negative effect on bowel preparation in outpatients, which might have been neglected for a long time. This work might help us draw attention to the importance of physician factors in improving the quality of bowel preparation among outpatients.

As shown in our study, the quality of bowel preparation between the two groups was significantly different, and the non-digestive physicians were identified to be a risk factor in the multivariate analysis. We speculate that the education on bowel preparation before the colonoscopy may be one of the most important reasons. Previous studies had explored the impact of education on the quality of bowel cleansing (20–22), which confirmed the effectiveness of patient education programs. Liu’s study (23) also revealed that inpatients under the care of nurses who underwent enhanced education on bowel preparation had a significantly better bowel preparation quality, and ward nurses’ education was the only independent risk factor, considering that the bowel preparation of inpatients was often guided by the nurses whereas that of outpatients was always guided by the referring physicians. We believed that verbal instructions accompanying written ones for outpatients seem to ameliorate the results. In addition, according to the European Society of Gastrointestinal Endoscopy (ESGE) Guideline (24), for patients undergoing morning colonoscopy, split-dose bowel preparation may be more suitable; however, for afternoon colonoscopy, a same-day regimen could be used as an acceptable alternative to split dosing. However, most non-digestive physicians lacked adequate instructions on bowel preparation and did not receive systematic training to perform colonoscopies. Therefore, they may be not as good as digestive physicians at adjusting the bowel preparation for patients’ characteristics. Reinforcing education among physicians not just on patients may also be a key point to improving bowel preparation of outpatients before a colonoscopy as ESGE recommended (24). However, we were limited to this retrospective real word data that did not quantify the education that was received by the physicians and patients on bowel preparation. Otherwise, we might be able to explore the reasons for the difference in bowel preparation between the two groups. Based on the current situation, the number of outpatients is large, and they will make an appointment with referring physicians who came from the different specialized departments according to the presence of various diseases. Therefore, further prospective research was needed to identify reliable reasons such as education level, the number of outpatient visits, and the salary level, for this difference caused by the non-digestive physicians.

For the other risk factor, BMI, our conclusions are consistent with a previous study (13), which was confirmed to be a critical role in predicting inadequate intestinal cleansing. However, we did not reach a consensus on comorbidities. The reason may be because we did not include comorbidities such as depression, previous diverticulitis, chronic constipation, but hyperlipidemia, and gout. In addition, our sample size for neurological diseases such as Parkinson’s disease or stroke/dementia was relatively small. Constipation is also a well-recognized risk factor for inadequate bowel preparation in patients (25), but we did not obtain a significant result, with a *P*-value of approximately 0.05 (*P* = 0.057) in the multivariate analysis. We think that this may be related to the limited sample size of this study and the insufficient sample size of patients with constipation, accounting for only 18.2%. On the other hand, previous abdominal surgery had also not been proven to be a risk factor, similar to other studies (14, 26). Other researchers who have searched for an explanation have indicated that only gastric/small intestinal surgery was a potential risk factor for poor bowel preparation (27), but we also included pelvic and gallbladder surgery. Low-residue diets were confirmed to be a protective factor in our results, which was consistent with previous research (28) and the recommendations by ESGE (3).

Our study is one of few that have confirmed the difference in the quality of bowel preparation between the digestive and non-digestive physicians and explored, from the physician’s perspective, a new predictor in suboptimal bowel preparation, which has not been reported in previous studies. Ignorance of this risk factor may be one reason why intestinal cleansing has remained unsatisfactory after many measures have been improved. This study might remind us that it is necessary to reinforce education not only on patients but also on physicians, help them flexibly change the doses or types of laxatives (29, 30), adopt a non–high-FODMAP diet (31, 32), and use of enhanced instructions for bowel preparation (33).

There were several limitations to this study. First, this was an observational study, and the availability and quality of data were limited, although all patients who missed critical information were eliminated. Second, this was a single-center study that involved only one hospital in China; therefore, we could not determine if there were differences between different hospitals, and the generalizability of the results is debatable. A multicenter study with a larger sample is needed to confirm our results. Third, we only analyzed outpatients and did not expand to inpatients for comprehensive analysis. However, we did not think that this was important for the results, because the bowel preparations of inpatients are mostly guided by nurses, which weakened the influence of referring physicians. Finally, we did find significant differences between the two groups of referring physicians, but the exact cause of the discrepancy between these groups is still unclear. We suspect that excessive specialization has led to a remarkably different focus on the disease, which has led non-digestive physicians to ignore the importance of colonoscopy and has affected patient compliance with bowel cleansing as a result. Therefore, we suggest that other aspects should be given more attention, for example, providing theoretical instruction for non-digestive physicians and helping them identify high-risk patients, together with providing a targeted type or volume of laxative, to improve this situation.

In conclusion, our study found that the quality of intestinal preparation by non-digestive physicians was inferior to that of digestive physicians. The findings that the non-digestive physician may be a risk factor will be directly relevant to a wide range of real-world outpatients. New ideas to improve the quality of bowel preparation from the perspective of physicians, not only patients, for colonoscopies in the future will be necessary.

## Data availability statement

The original contributions presented in the study are included in the article/supplementary materials. Further inquiries can be directed to the corresponding authors.

## Ethics statement

The studies involving human participants were reviewed and approved by the Ethics committee of the First hospital of Ningbo (NO.2020-R281). Written informed consent from the participants’ legal guardian/next of kin was not required to participate in this study in accordance with the national legislation and the institutional requirements.

## Author contributions

CL, CY, LX, XY, ZZ, and JX participated in the study’s conception and design. CY, LX, XY, HG, HL, JC, and WW provided the study materials and patients. CL, XY, HG, ZZ, JX, and WW collected data. CL, CY, HL, JC, and ZZ analyzed and explained the data. CL, CY, and LX participated in the manuscript writing. All authors contributed to manuscript revision, read, and approved the submitted version.

## Funding

This work was supported by the Medical Health Science and Technology Project of Zhejiang Provincial Health Commission (no. 2018KY681 to Hongpeng Lu) and the Xiangshan Science and Technology Plan Projects (no. 2020C6011 to Jian Chen).

## Acknowledgments

The authors thank the professional editors of AJE Company (www.aje.cn) for editing the English of this manuscript.

## Conflict of interest

The authors declare that the research was conducted in the absence of any commercial or financial relationships that could be construed as a potential conflict of interest.

## Publisher’s note

All claims expressed in this article are solely those of the authors and do not necessarily represent those of their affiliated organizations, or those of the publisher, the editors and the reviewers. Any product that may be evaluated in this article, or claim that may be made by its manufacturer, is not guaranteed or endorsed by the publisher.

## Abbreviations

ABP, adequate bowel preparation.
